# Dienogest increases the progesterone receptor isoform B/A ratio in patients with ovarian endometriosis

**DOI:** 10.1186/1757-2215-5-31

**Published:** 2012-11-01

**Authors:** Atsushi Hayashi, Akiko Tanabe, Sachiko Kawabe, Mika Hayashi, Hiroko Yuguchi, Yoshiki Yamashita, Kiyoji Okuda, Masahide Ohmichi

**Affiliations:** 1 Department of Obstetrics and Gynecology, Osaka Medical College, 2-7 Daigaku-machi, Takatsuki city, Osaka, 569-8686, Japan

**Keywords:** Dienogest, Progesterone receptor isoforms, Estrogen receptor isoforms, Ovarian endometriosis, Progesterone resistance

## Abstract

**Background:**

The resistance of endometriotic tissue to progesterone can be explained by alterations in the distribution of progesterone receptor (PR) and estrogen receptor (ER) isoforms. The aims of this study were to examine the expressions of PR-A, PR-B, ERα and ERβ in endometrioma and assess whether these expressions are affected by dienogest or leuprolide acetate (LA) treatment.

**Methods:**

We enrolled 60 females, including 43 patients with endometriosis (14 who received no medical treatment, 13 who received dienogest and 16 who received LA before undergoing laparoscopic surgery) and 17 patients with leiomyoma. The expression levels of PR and ER isoforms in eutopic and ectopic endometrium were assayed with quantitative real-time PCR, and confirmed with immunohistochemistry.

**Results:**

A decreased PR-B/PR-A ratio and an increased ERβ/ERα ratio were demonstrated in ectopic endometrium derived from females with endometriosis compared with the ratios observed in eutopic endometrium obtained from females without endometriosis. Although LA treatment did not affect the PR-B/PR-A and ERβ/ERα ratios, dienogest treatment increased the PR-B/PR-A ratio and decreased the ERβ/ERα ratio in patients with endometriomas.

**Conclusions:**

Dienogest may improve progesterone resistance in endometriotic tissue by increasing the relative expressions of PR-B and PR-A, and decreasing the relative expressions of ERβ and ERα.

## Background

Endometriosis is an estrogen-dependent inflammatory disease that affects 6-10% of females of reproductive age [1]. It is characterized by the presence of endometrium-like tissue outside the uterine cavity, primarily on the ovaries, and represents one of the most common causes of chronic pelvic pain, dysmenorrhea and infertility [2]. The main aims of treatment are to alleviate pain and other symptoms, reduce the size of the endometriotic lesions and improve the quality of life of affected individuals. Nonsteroidal anti-inflammatory drugs are frequently used by patients with endometriosis in an attempt to relieve pelvic pain, although clinical trial evidence to support the efficacy of these agents in endometriotic patients is lacking [3]. Gonadotropin-releasing hormone (GnRH) agonists are an established therapy for endometriosis. Although GnRH agonists provide effective pain relief and reduce the progression of endometriotic implants [4], the hypoestrogenic state they induce is associated with negative effects such as accelerated bone mineral density loss, hot flashes and vaginal dryness [5]. Oral contraceptives (OCs) are widely used to treat the symptoms of endometriosis, although they are not approved for this indication in the majority of countries due to the lack of supportive trial evidence [6]. Progestins have long been used for the treatment of endometriosis to relieve pain by suppressing ovarian estrogen biosynthesis, in turn, suppressing growth and inflammation [7]. Unfortunately, the relief of pain appears to be relatively short-term [8], and approximately 9% of females with endometriosis simply do not respond to progestin therapy due to unknown reasons [9]. In fact, a general tendency for relative progesterone resistance within the eutopic and ectopic endometrium of females with endometriosis is well-documented [1,10].

Recently, two major progesterone receptor (PR) isoforms were identified, namely PR-A and PR-B (11). PR-A is a 94-kDa protein, whereas PR-B is a 114-kDa protein that contains an additional NH2-terminal stretch of approximately 165 amino acids containing a region encoding a transactivation function. These isoforms may arise as a result of either initiation of translation from alternative sites in the same messenger RNA (mRNA) [11] or by transcription from alternative promoters [12]. Although the exact functions of each of these isoforms remain unclear, there is increasing evidence that they are functionally different [12,13]. PR-B tends to be a stronger activator of progesterone target genes, whereas PR-A has been shown to act as a dominant repressor of PR-B [14,15].

Endometriosis is associated with a reduced response to progesterone in both eutopic and ectopic endometrium. According to recent reports, the resistance of endometriotic tissue to progesterone, evident in both laboratory and clinical observations, can be explained by alterations in the distribution of ER and PR isoforms and dysregulation of progesterone target genes [10,16-18]. Because the effects of progesterone on target genes are conferred primarily by PR-B in the endometrium [19], the presence of the inhibitor isoform-A and the absence of the stimulatory isoform-B provide a possible explanation for progesterone resistance in endometriotic implants. In fact, a decreased PR-B/PR-A ratio has been demonstrated in ectopic tissue [20,21], and recent reports suggest that the tendency toward progesterone resistance in patients with endometriosis is likely the result of the promotion of hypermethylation of PR-B, which renders PR-B either silenced or downregulated [21]. Moreover, a number of investigators have reported markedly elevated levels of ERβ and lower levels of ERα in human endometriotic tissues and primary stromal cells compared with that observed in eutopic endometrial tissues and cells [16,22,23]. ERβ, acting as an ERα suppressor, might contribute to the decreased PR levels and progesterone resistance observed in patients with endometriosis [24].

Dienogest (17a-cyanomethyl-17b-hydroxyestra-4,9-dien-3-one) is an oral progestin that has been systematically investigated for the treatment of endometriosis in dose-ranging [25], placebo-controlled [26,27], active comparator-controlled [28,29] and long-term trials [30] conducted in Europe and Japan. The main anti-endometriotic effect of dienogest has been suggested to be attributable to central inhibition of ovulation. Furthermore, direct antiproliferative effects of dienogest have been demonstrated in human eutopic endometrial stromal cells [31]. Recent studies demonstrate that dienogest inhibits the proliferation of endometriotic stromal cells [32], prostaglandin E2 production and the aromatase mRNA expression of the endometrial epithelial cell line [33]. However, there is no evidence regarding whether dienogest improves progesterone resistance in patients with endometriosis. In the present study, therefore, we examined the effects of dienogest on alterations in the ratios of PR-B to PR-A and ERβ to ERα in ectopic endometrial tissue obtained from endometriotic females.

## Methods

### Patients and tissue collection

Sixty patients treated between January 2002 and July 2010 were included in this study. All patients were under treatment at the department of obstetrics and gynecology of Osaka Medical College. This was a retrospective cross-sectional case-controlled study of human tissue samples approved by the Institutional Review Board of Osaka Medical College. Written informed consent was obtained from all patients participating in the study.

The inclusion criteria were: older than 20 years age of and no more than 50 years of age at the time of the surgical procedure, the presence of regular menstrual cycles (24–35 days of interval) with the exception of those treated with leuprolide acetate (Leuplin; Takeda pharmaceutical, Osaka, Japan) for endometriosis, the absence of any evidence of past or recent pelvic inflammatory disease and no history of any hormonal treatment for at least 12 months at baseline. Trans-vaginal ultrasonography was performed for all patients, and showed mainly hypoechoic cystic masses in the ovaries, and the presence of ovarian endometriomas were confirmed before surgery by magnetic resonance imaging (MRI), which showed high-intensity areas on both T1- and T2-weighted images. The serum level of CA125 was also measured before surgery using automated assays on a Roche Modular E170 instrument (Roche, Vilvoorde, Belgium) at the central laboratories of Osaka Medical College. Tissue specimens were obtained from females (n=43) treated with dienogest at a dose of 2 mg (dienogest group; n=13) for three to five months or leuprolide acetate (LA) at a dose of 3.75 mg (LA group; n=16) administered before surgery. Simultaneous sampling of ovarian endometrioma capsules was performed during laparoscopic surgery for indications of adnexal masses consistent with ovarian endometrioma. The stage of endometriosis in each case was documented according to the revised American Society of Reproductive Medicine Criteria (r-AFS stage) [34]. The diagnosis of endometriosis was confirmed histologically. Normal endometrial tissues (n=17) were obtained using biopsies during the proliferative phase of the menstrual cycle in patients undergoing hysterectomy for uterine fibroids.

The samples obtained from the ectopic and eutopic endometrium were immediately frozen in liquid nitrogen for further RT-PCR analyses, fixed in 10% formaldehyde and then routinely processed for paraffin embedding for a histological analysis.

### OneStep real-time polymerase chain reaction

Total RNA was obtained using the RNeasy Mini kit (Qiagen, Germantown MD), and 2μg were reverse transcribed with Superscript II RNase H-reverse transcriptase (Invitrogen) using random primers according to the manufacturer’s instructions. Oligonucleotide primers for TaqMan probes were designed with the use of Primer Express (version 1.0; Perkin-Elmer Applied Biosystems, Tokyo, Japan) from the GeneBank database. Human GAPDH was purchased from Perkin-Elmer Applied Biosystems and used as an internal standard. The primers used in this study are shown in Table 1. The first primer set, termed PR-B, was designed to amplify sequences specific for PR-B (upstream of the second AUG translation initiation site), whereas the second primer set, total PR, was designed to amplify sequences downstream of the second AUG translation initiation site. None of these primer sets corresponded to sequences in any of the other steroid hormone receptors. The cDNA template was amplified using quantitative real-time polymerase chain reaction (qRT-PCR), as previously described [35]. Briefly, the cDNA template was amplified in a 20 μL reaction containing 1 x TaqMan Universal PCR Master Mix (Perkin-Elmer Applied Biosystems), 200 nM forward and reverse primers and 100 nM TaqMan probe. The TaqMan PCR conditions were: 95°C for 15 seconds followed by 60°C for one minute for 45 cycles in each case on OneStep real-time PCR (Perkin-Elmer Applied Biosystems). The amplification of the target gene mRNA relative to GAPDH was compared using the ΔΔCt method. The abundance of PR-A was calculated by subtracting the relative abundance of PR-B from that of total PR. 

**Table 1:** Primers used for TaqMan real-time polymerase chain reaction

**Gene**	**Primer**
PR-B	5^′^-CTGGCCTATCCTGCCTGCCTCA-3^′^
	5^′^-TGTCCAAGACACTGTCCAGCAG-3^′^
total PR	5^′^CGTGCCTATCCTGCCTCTA-3
	5^′^CCGCCGTCGTAACTTTCGT-3^′^
ERα	5^′^-GGGAAGCTACTGTTTGCTCCTAAC-3^′^
	5^′^-CACCATGCCCTTACACATTC-3^′^
ERβ	5^′^-GATCGCTAGAACACACCTTACCTGTA-3^′^
	5^′^-GCGCAACGGTTCCCACTA-3^′^

### Histology and immunohistochemistry

All specimens fixed in 10% paraformaldehyde solution were embedded in paraffin blocks. The sections were stained with hematoxylin and eosin for histological evaluation of the tissues. For deection of PR-A, a human PR-A-specific mouse monoclonal antibody purchased from Novocastra (NCL-L-PGR-312; Vision BioSystems Inc., Norwell, MA) was used [36,37]. For PR-B immunostaining, mouse monoclonal antibody Ab-6 (NeoMarkers, Fremont, CA) was used [36,38]. For ERα and ERβ immunostaining, rabbit polyclonal antibodies (LS-B1470 and LS-B945, respectively, LifeSpan Biosciences, Seattle, WA, USA) were used. The antigen-antibody complexes were identified using the Universal DAKO LSAB2-labeled streptavidin-biotin peroxidase kit (Lifespan Biosciences).

### Statistics

All experiments were performed in triplicate. The statistical calculations were performed using the StatView statistical software package (SAS Institute, Cary, NC), and the statistical significance of each difference was determined using the Kruskal-Wallis and Mann-Whitney U test or paired *t*-test as appropriate. A *P* value of < 0.05 was considered to be statistically significant.

## Results

### Patient characteristics

A total of 14 females who did not receive any medical treatment, 13 females who received dienogest and 16 females who received LA were evaluated in this study (Table 2). There were no relevant group differences in age or VAS at baseline. The use of concomitant medications recorded in patient-maintained diaries, including analgesic medications for endometriosis, did not differ relevantly between the groups at baseline.

**Table 2:** Baseline patient characteristics

	**Patients with uterine fibroids (n=17)**	**Endometriosis**				
		**Control (n=14)**	**Dienogest 2 mg (n=13)**		**LA 3.75 mg (n=16)**	
	**baseline**	**baseline**	**baseline**	**preoperation**	**baseline**	**preoperation**
Age (years, mean ± SD	44.2 ± 6.8	34.9 ± 8.1	34.3 ± 5.7		37.5 ± 7.2	
Duration of drug administration	-	-	21.6 ± 11.5		12.6 ± 2.8	
Pelvic pain VAS (mm, mean ± SD)	23.8 ± 18.2	53.1 ± 29.9	46.2 ± 22.6^a^	20.6 ± 15.1^b^	56.3 ± 41.0^ab^	21.1 ± 18.0^b^
CA-125 (U/ml, mean ± SD)	33.1 ± 20.4	60.3 ± 34.1	60.6 ± 35.8	34.8 ± 14.9^c^	52.9 ± 42.8	34.9 ± 20.8
r-AFS stage (n,%)						
I: minimal	10 (58%)	0 (0%)	0 (0%)		0 (0%)	
II: mild	3 (18%)	0 (0%)	0 (0%)		2 (13%)	
III: moderate	4 (24%)	5 (36%)	8 (62%)		9 (56%)	
IV: severe	0 (0%)	9 (64%)	5 (38%)		5 (31%)	

LA, leuprolide acetate; VAS, visual analogue scale; r-AFS, revised American Fertility Society.

SD, standard deviation.

A: *p* <0.01 compared to patients with uterine fibroids, b: *p* <0.01 compared to endometriosis-control, c: *p* <0.05 compared to baseline.

At baseline, the mean ± SD VAS score was 53.1 ± 29.9 mm in the endometriosis-control group, 46.2 ± 22.6 mm in the endometriosis-dienogest group and 56.3 ± 41.0 mm in the LA group. Following surgical treatment, the mean VAS score decreased to 20.6 ± 15.1 mm in the dienogest group and 21.1 ± 18.0 mm in the LA group, demonstrating the non-inferiority of dienogest versus LA as measured by observed VAS score changes. At baseline, the mean ± SD serum CA125 level was 60.3 ± 34.1 U/ml in the endometriosis-control group, 60.6 ± 35.8 U/ml in the endometiosis-dienogest group and 52.9 ± 42.8 U/ml in the LA group. Following surgical treatment, the mean serum CA125 level significantly decreased to 34.8 ± 14.9 U/ml in the dienogest group and to 34.9 ± 20.8 U/ml in the LA group.

### PR isoform expression and PR-B/PR-a ratios in eutopic and ectopic endometrium

Previous studies have demonstrated that the expression of repressive PR-A and the apparent downregulation of stimulatory PR-B may explain the development of progesterone resistance in patients with endometriosis [1,39]. This study investigated whether treatment with dienogest or leuprolide acetate attenuates the expression of PR isoforms in ectopic endometrium.

**Figure 1:**
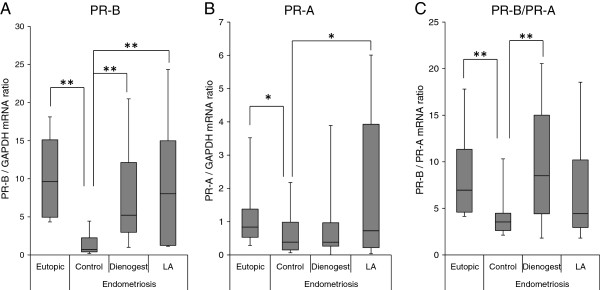
**PRs expression and PR-B/PR-A ratios in eutopic/ectopic endometrium following treatment with dienogest or leuprolide acetate.** Total RNA was isolated from eutopic endometrial samples (Eutopic, n=17) obtained with biopsies performed during the proliferative phase of the menstrual cycle in patients undergoing hysterectomy for uterine fibroids. Total RNA was also isolated from endometriotic samples obtained from females who did not receive medical treatment (Control, n=14) and endometriotic samples obtained from females treated with dienogest at a dose of 2 mg (Dienogest, n=13) or leuprolide acetate (LA, n=16). The total RNA was then reverse-transcribed. **A**: The relative expression ratio of the PR-B gene was calculated in comparison to the GAPDH expression. **B**: The relative expression ratio of the PR-A gene was calculated by subtracting the relative abundance of PR-B from that of total PR. **C**: For each experimental sample, the data are graphically illustrated as the ratio between the relative expressions of PR-B and PR-A. The center lines indicate the median ratio. The columns and vertical bars indicate the 25–75 percentiles. Significant differences are indicated by an asterisk. *p<0.05, **p<0.01.

The expression of PR-B (Figure 1A) was significantly dysregulated in control ectopic endometrial samples obtained from females who did not receive any treatment compared with that observed in the eutopic endometrium of patients with fibroids. In contrast, the endometrioma samples obtained from the patients who received dienogest or LA treatment showed a significantly greater expression of PR-B compared with that observed in the control tissues. The expression of PR-A (Figure 1B) was also significantly decreased in control ectopic endometrial samples compared with that observed in eutopic endometrium. Although the endometrioma samples obtained from the patients who received LA treatment showed a significantly greater expression of PR-A, dienogest treatment did not alter the expression of PR-A. Typical mRNA expression patterns of the PR isoforms are shown in the Additional file 1: Figure A, and explained in the Additional file 2. Progesterone resistance may be explained by the absence of PR-B and the dominant expression of PR-A [20,21]; therefore, we analyzed the PR-B/PR-A ratio at the mRNA level (Figure 1C). In the control ectopic endometrial samples, the PR-B/PR-A ratio was significantly decreased compared with that observed in eutopic endometrium. Interestingly, dienogest treatment improved the PR-B/PR-A ratio; however, there were no significant differences in endometrioma after LA treatment. 

### ER isoform expression and ERβ/ERα ratios in eutopic and ectopic endometrium

This study investigated whether treatment with dienogest or leuprolide acetate attenuates the expression of ER isoforms in ectopic endometrium.

**Figure 2:**
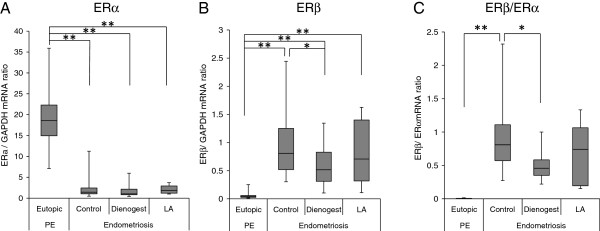
**ERs expression and ERβ/ERα ratios in eutopic/ectopic endometrium following treatment with dienogest or leuprolide acetate.** Total RNA was isolated from eutopic endometrial samples (Eutopic, n=17) obtained with biopsies performed during the proliferative phase of the menstrual cycle in patients undergoing hysterectomy for uterine fibroids. Total RNA was also isolated from endometriotic samples obtained from females who did not receive medical treatment (Control, n=14) and endometriotic samples obtained from females treated with dienogest at a dose of 2 mg (Dienogest, n=13) or leuprolide acetate (LA, n=16). The total RNA was then reverse-transcribed. **A**: The relative expression ratio of the ERα gene was calculated in comparison to the GAPDH expression. **B**: The relative expression ratio of the ERβ gene was calculated in comparison to the GAPDH expression. **C**: For each experimental sample, the data are graphically illustrated as the ratio between the relative expressions of ERβ and ERα. The center lines indicate the median ratio. The columns and vertical bars indicate the 25–75 percentiles. Significant differences are indicated by an asterisk. *p<0.05, **p<0.01.

The expression of ERα (Figure 2A) was significantly dysregulated in the control ectopic endometrioma samples obtained from females who did not receive any treatment compared with that observed in the eutopic endometrium of patients with fibroids. In addition, the endometrioma samples obtained from the patients who received dienogest or LA treatment showed a significantly lower expression of ERα compared with that observed in eutopic endometrium. The expression of ERβ (Figure 2B) was also significantly elevated in the control ectopic endometrial samples compared with that observed in eutopic endometrium. Dienogest, but not LA, treatment altered the expression of ERβ. Typical mRNA expression pattern of the ER isoforms are shown in the Additional file 1: Figure B, and explained in Additional file 2. A number of investigators have reported markedly elevated levels of ERβ and lower levels of ERα in human endometriotic tissues compared with that observed in eutopic endometrial tissues and cells [16,22,23]. Therefore, we also analyzed the ERβ/ERα ratio at the mRNA level (Figure 2C). In the control ectopic endometrial samples, the ERβ/ERα ratio was significantly increased compared with that observed in eutopic endometrium. Interestingly, dienogest treatment significantly improved the ERβ/ERα ratio; however, there were no significant differences in endometrioma after LA treatment. 

### Expression of PR and ER isoform proteins in the eutopic and ectopic endometriotic samples

**Figure 3:**
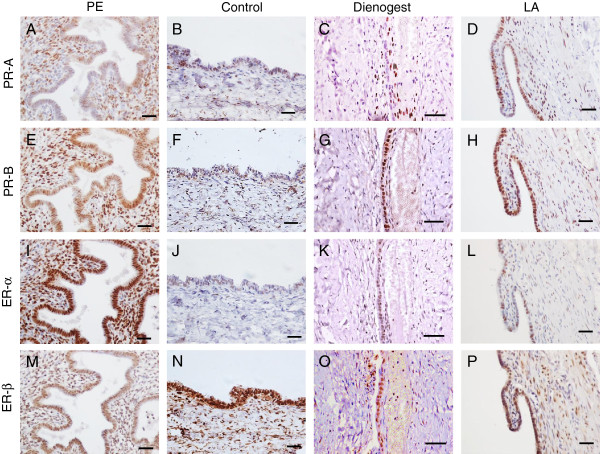
**Typical example of PR and ER isoform expression in patients with ovarian endometriosis.** PR-A and PR-B were detected using PR-A (**A**, **B**, **S**, **D**) and PR-B (**E**, **F**, **G**, **H**) antibodies. ERα and ERβ were detected using ERα (**I**, **J**, **K**, **L**) and ERβ (**M**, **N**, **O**, **P**) antibodies. The eutopic endometrium (**A**, **E**, **I**, **M**) in the proliferative phase was obtained from females undergoing hysterectomy for uterine fibroids. The ectopic endometrium was obtained from endometrioma of the control females (**B**, **F**, **J**, **N**), dienogest-treated females (**C**, **G**, **K**, **O**) and LA-treated females (**D**, **H**, **L**, **P**). Scale bar: 25 m.

An immunohistochemical analysis revealed that PR-B immunoreactivity was strongly localized to the glandular epithelium and stromal cells in the eutopic endometrial samples during the proliferative phase (Figure 3E) and faintly localized to the glandular epithelium in the control ectopic endometrium (Figure 3F). In contrast, PR-A immunoreactivity appeared faintly in both the eutopic endometrial samples (Figure 3A) and the control ectopic endometrium (Figure 3B). After the patients were treated with dienogest or LA, immunostaining of PR-B increased in the ectopic endometrial epithelium (Figure 3G and H). ERα was strongly localized to the glandular epithelium and stromal cells in the eutopic endometrium (Figure 3I) and faintly localized in the control ectopic endometrium (Figure 3J). Dienogest and LA treatment had no effect on the ERα expression (Figure 3K and L). ERβ immunoreactivity appeared faintly in the eutopic endometrial samples (Figure 3M). In contrast, ERβ was strongly localized to the epithelium and stromal cells in the ectopic endometrium (Figure 3N). Although dienogest treatment decreased the ERβ expression in ectopic endometrioma (Figure 3O), LA treatment seemed to have no effect on the ERβ expression (Figure 3P). No immunostaining was found in eutopic or ectopic endometrium when the primary antibodies were omitted (data not shown).

## Discussion

The current study demonstrated statistically significant decreases in both PR–B and PR-A messenger RNA and proteins in ectopic endometrium derived from females with endometrioma who did not receive any medical treatment (Figure 1A, 1B, 3B, and 3F). Furthermore, the relative expressions of PR-B and PR-A were significantly lower in ectopic endometrium (Figure 1C). According to Attia et al. [20], the resistance of endometriotic tissue to progesterone, evident in both laboratory and clinical observations, can be explained by the absence of PR-B transcripts and proteins and the presence of PR-A in ectopic lesions. Similar findings have been reported in epithelial cells selected from a small number of ectopic samples [10]. Recently, independent investigators suggested that alterations in the relative expressions of PR-A and PR-B in endometrial cells may also play a pivotal role in the pathogenesis of endometriosis. A decreased PR-B/PR-A ratio has been demonstrated in ectopic tissue [5,10], and our findings are consistent with the results of these studies.

Several investigators have reported markedly higher levels of ERβ and lower levels of ERα in human endometriotic tissues and primary stromal cells compared with that observed in eutopic endometrial tissues and cells [22,40]. Recently, Bulun [41] reported that the levels of the nuclear receptors ERα, ERβ and PR are quite different in endometriotic tissue and endometrium. The high ERβ/ ERα ratiosin endometriotic stromal cells in turn lead to increased ERβ binding to the PR promoter and mediate downregulation of the expression of PR [21]. ERβ acts as a suppressor of ERα in both endometrial and endometriotic stromal cells by binding to regulatory elements of specific promoters of the ERα and PR genes [42]. Therefore, ERα deficiency in endometriotic patients may be responsible for the failure of E2 to induce the PR expression, thus contributing to secondary PR deficiency and progesterone resistance in females with endometriosis. Although strikingly high quantities of E2 produced via local aromatase activity are observed in endometriotic tissue [43,44], the E2-dependent induction of PR is strongly inhibited [20]. Findings consistent with these studies were observed in the current study, which demonstrated statistically significant higher levels of ERβ and lower levels of ERα in ectopic endometrium derived from females with endometrioma (Figure 2 and Figure 3).

The endometrioma samples obtained from the patients who received dienogest or LA treatment showed a significantly higher expression of PR-B compared with that observed in the control endometrioma samples (Figure 1A and Figure 3). Although LA treatment increased the PR-A expression in the endometrioma samples, dienogest treatment did not alter the expression of PR-A (Figure 1B). Consequently, dienogest treatment improved the PR-B/PR-A ratio; however, there were no significant differences in endometrioma after LA treatment (Figure 1C). Progesterone has recently been shown to reverse E2-stimulated increases in the ERβ mRNA and protein expression in cultured hippocampal slices [45]. Our current study demonstrates that dienogest treatment alters the expression of ERβ (Figure 2B and Figure 3) and significantly decreases the ERβ/ERα ratio in endometrioma (Figure 2C). A recent report demonstrated that the promoter region of PR-B, but not PR-A, is hypermethylated in patients with endometriosis [21]. Although further studies are needed to clarify whether medical treatments might be associated with the methylation status of the PR-B promoter and to explore the mechanisms underlying the ERβ downregulation induced by dienogest, a decreased ERβ/ERα ratio in the endometriotic tissues of patients treated with dienogest may be responsible for the observed improvements in the PR expressions.

## Conclusions

We demonstrated that dienogest improves progesterone resistance in endometrial tissue. This finding enhances understanding of the anti-endometriotic effects of dienogest. It is possible that PR-B deficiency is only the tip of the iceberg with regard to the pathogenesis of endometriosis and that numerous other molecular aberrations may also contribute to the development of resistance to hormone treatments in females with endometriosis. Although our findings may explain at least part of the mechanisms underlying the clinical improvements observed in endometriotic patients using dienogest, the normalization of the PR expression profile observed in this study suggests that dienogest may be an effective and long-term treatment for endometriosis.

## Competing interests

The authors declare that they have no competing interests.

## Authors’ contributions

All authors read and approved the final manuscript.

## Supplementary Material

Additional file 1 **Typical mRNA expression patterns of the PR and ER isoforms.** A: A representative agarose gel showing amplicons of PR-B (upper panel), total-PR (middle panel), and β-actin (lower panel). B: A representative agarose gel showing amplicons of ERα (upper panel), ERβ (middle panel), and β-actin (lower panel). Click here for file

Additional file 2 Supplemental Results. Click here for file
