# Supplementary material for: Dienogest increases the progesterone receptor isoform B/A ratio in patients with ovarian endometriosis
Source: J Ovarian Res. 2012 Nov 1;5:31. doi: 10.1186/1757-2215-5-31 (PMC3541078; doi:10.1186/1757-2215-5-31)
Supplement: Additional file 1 — Typical mRNA expression patterns of the PR and ER isoforms. A: A representative agarose gel showing amplicons of PR-B (upper panel), total-PR (middle panel), and β-actin (lower panel). B: A representative agarose gel showing amplicons of ERα (upper panel), ERβ (middle panel), and β-actin (lower panel). [file 1757-2215-5-31-S1.pptx]

## Slide 1
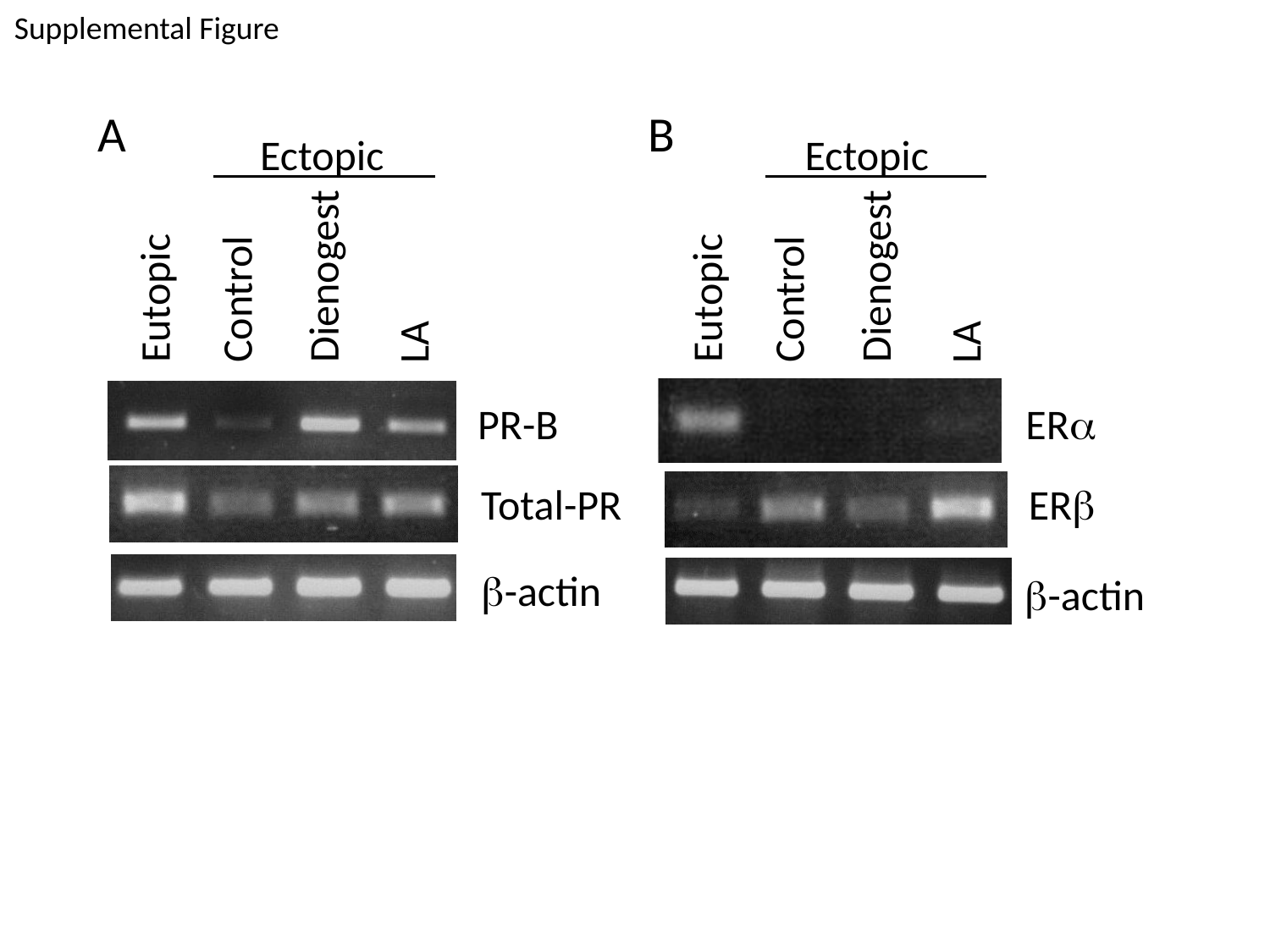

Supplemental Figure
A
B
Ectopic
Ectopic
Dienogest
Dienogest
Eutopic
Eutopic
Control
Control
LA
LA
ERa
PR-B
ERb
Total-PR
b-actin
b-actin
