# Supplementary material for: Dienogest increases the progesterone receptor isoform B/A ratio in patients with ovarian endometriosis
Source: J Ovarian Res. 2012 Nov 1;5:31. doi: 10.1186/1757-2215-5-31 (PMC3541078; doi:10.1186/1757-2215-5-31)
Supplement: Additional file 2 — Supplemental Results. [file 1757-2215-5-31-S2.doc]

**Supplemental Results**

The expression of PR-B (Suppl. A, upper panel) was significantly dysregulated in control ectopic endometrial samples obtained from females who did not receive any treatment compared with that observed in the eutopic endometrium of patients with fibroid tumors. In contrast, the endometrioma samples obtained from the patients who received dienogest treatment showed a significantly greater expression of PR-B compared with that observed in the control tissues. However, in LA group, the PR-B expression was not as high as that obtained from the samples in the dienogest group.

The second primer set, total-PR, was designed to amplify sequences downstream of the second AUG translation initiation site of the PR coding gene. Therefore, representative agarose gels are presented showing the total-PR mRNA expression. The expression of total-PR (Suppl. A, middle panel) was also significantly decreased in control ectopic endometrial samples compared with that observed in eutopic endometrium. The total-PR expression in the dienogest group was as low as that obtained from the LA group.

The expression of ERα (Suppl. B, upper panel) significantly decreased in the ectopic endometrium obtained from the control, dienogest and LA groups. ERβ  mRNA was highly expressed in the control endometrioma tissue samples (Suppl. B, middle panel). Dienogest treatment decreased the ERβ expression, however, LA treatment did not alter the ERβ expression in the ectopic endometrium.

**Supplemental Figure Legend**

**Supplemental Figure. Typical mRNA expression patterns of the PR and ER isoforms**

A: A representative agarose gel showing amplicons of PR-B (upper panel), total-PR (middle panel), and β-actin (lower panel). B: A representative agarose gel showing amplicons of ERα (upper panel), ERβ (middle panel), and β-actin (lower panel).
